# Using imperfect data in predictive mapping of vectors: a regional example of *Ixodes ricinus* distribution

**DOI:** 10.1186/s13071-019-3784-1

**Published:** 2019-11-14

**Authors:** Rita Ribeiro, Jude I. Eze, Lucy Gilbert, G. R. William Wint, George Gunn, Alastair Macrae, Jolyon M. Medlock, Harriet Auty

**Affiliations:** 1 Department of Veterinary and Animal Science, Northern Faculty Scotland’s Rural College, An Lòchran, 10 Inverness Campus, Inverness, IV2 5NA UK; 2 The Royal (Dick) School of Veterinary Studies and the Roslin Institute, University of Edinburgh, Easter Bush Campus, Midlothian, EH25 9RG UK; 3 Biomathematics and Statistics Scotland, JCMB, The King’s Buildings, Peter Guthrie Tait Road, Edinburgh, EH9 3FD UK; 4 Institute of Biodiversity, Animal Health and Comparative Medicine, University of Glasgow, Glasgow, G12 8QQ UK; 5 Environmental Research Group Oxford, c/o Department of Zoology, South Parks Road, Oxford, OX1 3PS UK; 6 Medical Entomology & Zoonoses Ecology, Emergency Response Department-Science & Technology, Public Health England (PHE), Porton Down, Salisbury, SP4 0JG UK

**Keywords:** Data quality, Decision making, *Ixodes ricinus*, Predictive maps, Public health, Uncertainty, Vector-borne diseases

## Abstract

**Background:**

Knowledge of *Ixodes ricinus* tick distribution is critical for surveillance and risk management of transmissible tick-borne diseases such as Lyme borreliosis. However, as the ecology of *I. ricinus* is complex, and robust long-term geographically extensive distribution tick data are limited, mapping often relies on datasets collected for other purposes. We compared the modelled distributions derived from three datasets with information on *I. ricinus* distribution (quantitative *I. ricinus* count data from scientific surveys; *I. ricinus* presence-only data from public submissions; and a combined *I. ricinus* dataset from multiple sources) to assess which could be reliably used to inform Public Health strategy. The outputs also illustrate the strengths and limitations of these three types of data, which are commonly used in mapping tick distributions.

**Methods:**

Using the Integrated Nested Laplace algorithm we predicted *I. ricinus* abundance and presence–absence in Scotland and tested the robustness of the predictions, accounting for errors and uncertainty.

**Results:**

All models fitted the data well and the covariate predictors for *I. ricinus* distribution, i.e. deer presence, temperature, habitat, index of vegetation, were as expected. Differences in the spatial trend of *I. ricinus* distribution were evident between the three predictive maps. Uncertainties in the spatial models resulted from inherent characteristics of the datasets, particularly the number of data points, and coverage over the covariate range used in making the predictions.

**Conclusions:**

Quantitative *I. ricinus* data from scientific surveys are usually considered to be gold standard data and we recommend their use where high data coverage can be achieved. However in this study their value was limited by poor data coverage. Combined datasets with *I. ricinus* distribution data from multiple sources are valuable in addressing issues of low coverage and this dataset produced the most appropriate map for national scale decision-making in Scotland. When mapping vector distributions for public-health decision making, model uncertainties and limitations of extrapolation need to be considered; these are often not included in published vector distribution maps. Further development of tools to better assess uncertainties in the models and predictions are necessary to allow more informed interpretation of distribution maps.
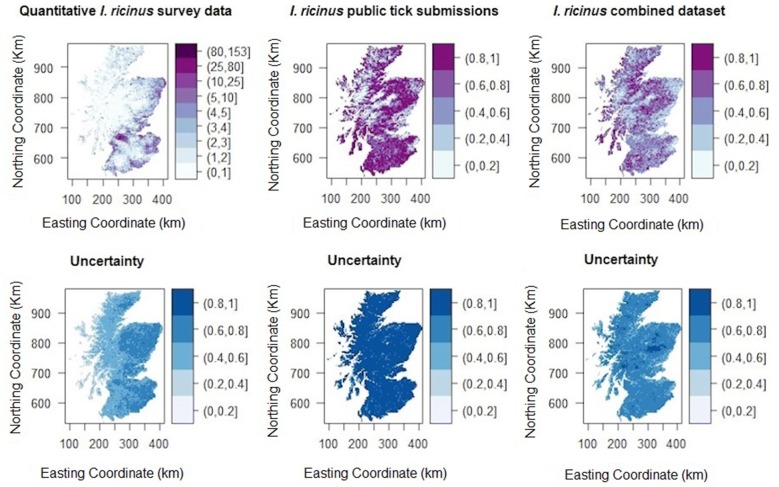

## Background

Tick-borne diseases affect the health of both humans and animals and impact on the economy [1]. Lyme borreliosis, caused by the *Borrelia burgdorferi* (*sensu lato*) complex of bacteria, is the most prevalent arthropod-borne disease of humans in the Northern Hemisphere [2]. Since the early 1990s, the number of reported cases of Lyme borreliosis is rising, and the geographical distribution of cases is expanding [3]. One of the reasons for these shifts is the expansion of the distribution of the main vector of *B. burgdorferi* in Europe, the tick *Ixodes ricinus* [4].

*Ixodes ricinus* is the most abundant and widespread tick species in western Europe. As well as *Borrelia burgdorferi* (*s.l.*) [5], it transmits other pathogens responsible for causing diseases of humans and animals. This species is now found at higher northern latitudes and higher altitudes than previously reported [6, 7] and is more abundant in several areas [8, 9]. The expansion of *I. ricinus* could be partly due to changes in host animal availability such as an increase in deer abundance and distribution [10], since deer densities frequently correlate with tick abundance [11] and perhaps also partly due to abiotic environmental changes, such as climate warming, since higher temperatures can increase interstadial development rate, oviposition rate and egg development rates [12], and the proportion of active ticks [13, 14].

Understanding the drivers of the distribution and abundance of *I. ricinus* is one of the critical steps in assessing the risk of tick-borne diseases and informing policy on awareness and control strategies [15]. Reliable maps of *I. ricinus* distribution are essential to understand and identify changes in the pattern of *I. ricinus* and diseases it transmits [16], and to identify hot-spots of vector occurrence that will inform policy makers in allocating resources to high risk areas, including targeting education and preventive measures [3] or management of important tick population hosts as deer [11].

Several *I. ricinus* distribution models and maps have been published, aiming to predict current and future distribution of *I. ricinus* on different geographic scales, ranging from European to country or local levels [17–20]. The purpose of the study will determine the geographical scale of the map and the resolution will determine the degree of precision, realism and applicability of the models and maps [21]. Therefore, if the objective is to make decisions at country or regional levels, finer resolution maps can detect high variability in tick distribution patterns.

However, predicting *I. ricinus* distribution and abundance is challenging due to the complex ecology of *I. ricinus* (with multiple tick stages and multiple hosts), the limited availability of detailed, long-term and geographically extensive tick distribution data, and a wide range of environmental variables that may influence tick distribution. Reliable data on *I. ricinus* presence and absence or abundance can be collected during field surveys which use standardized sampling methods, such as the blanket-dragging technique [22]. However, the resources required for field sampling (trained personnel, cost and time required) mean that data are often not available at meaningful spatial and seasonal scales [17]. Other sources of data that were not collected with the purpose of predictive mapping are therefore often used instead. Data submitted by the public can be used to improve the knowledge of *I. ricinus* distribution [23] but usually comprise presence-only data so are subject to biases. An alternative approach, often undertaken by large-scale projects such as VectorNet [22], is to combine available data sources into one composite dataset.

Although Lyme borreliosis is an important public health concern in Scotland [24], published predictive maps of *I. ricinus* distribution in Scotland are limited, particularly at an appropriate scale for national and local decision-making. Although some (as yet unpublished) predictive maps have been made [25, 26], the only peer reviewed publication is a mechanistic model predicting the distribution of infected *I. ricinus* nymphs now and under climate warming [17]. Large-scale presence–absence maps at the European level [27] do not have sufficient resolution for targeting public health resources within Scotland, where *I. ricinus* is endemic.

The main aim of this study was to compare the performance of three datasets to predict *I. ricinus* distribution in Scotland, in order to produce predictive maps for use by decision-makers. We generated model, map and uncertainty outputs of predicted tick abundance and distribution over Scotland from three datasets: (i) quantitative (abundance) *I. ricinus* data from scientific surveys; (ii) *I. ricinus* presence-only data resulted from public submissions plus absence points; and (iii) a composite dataset that combines presence data from public submissions, presence and absence from scientific tick surveys, literature reviews and expert opinion and, absence from a habitat suitability mask for *I. ricinus*. These datasets, which comprise the only data available on tick distributions at a national scale for Scotland, also represent three data types commonly used in mapping tick distributions (i.e. surveyed abundance; surveyed presence and absence; and public submission). We assessed the outputs derived from these different inputs to highlight the strengths and limitations of each data type, and compared the performance of these different types of data in predicting tick distribution, in order to make recommendations for future tick mapping for use in a Public Health context.

## Methods

### Tick data

We used three datasets with information on *I. ricinus* occurrence or abundance in Scotland. As is often the case with predictive mapping exercises, none of these datasets were collected with the main objective of predicting tick distribution at the national level, but they represent the most extensive datasets currently available for mainland Scotland.

#### Dataset 1: “quantitative I. ricinus survey data”

**Fig. 1:**
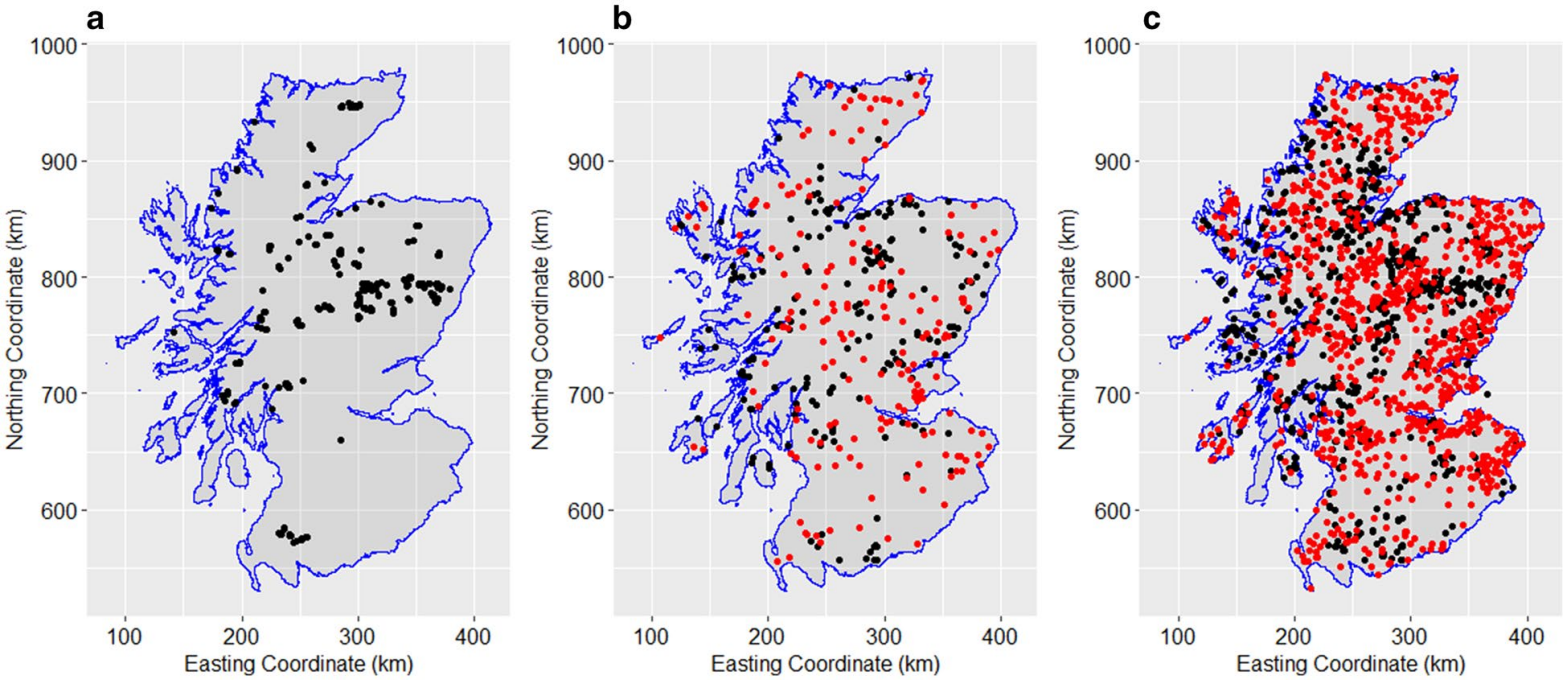
**a** Distribution of sites of tick quantitative field surveys in mainland Scotland (Dataset 1). **b** Distribution of sites of presence-only reports (black dots) and absences of *I. ricinus* (red dots) (Dataset 2). **c** Distribution of combined presence of *I. ricinus* from field surveys and public submissions (black) and absences (red dots) (Dataset 3)

Dataset 1 (Fig. 1a) is quantitative tick data, and consists of counts of questing *I. ricinus* ticks (nymphs and adults) in sampled environments in mainland Scotland between 2006 and 2017. Questing ticks were sampled using the standard technique of dragging a white blanket of 1 m^2^ across the ground vegetation area of 10 × 10 m, with an average of approximately 15 drags per site [28–30]. During this 11-year period, 687 sites were visited, with varying frequency (1–4 visits), and a total of 10,611 drags were performed.

#### Dataset 2: “I. ricinus public tick submissions”

Dataset 2 (Fig. 1b) comprised tick submissions by the public to Public Heath England (PHE) made through the Tick Surveillance Scheme between 1998 and 2016 in mainland Scotland. The scheme focuses on tick surveillance in England and Wales; ticks are also submitted from Scotland, but in relatively small numbers. The number of ticks (adults and nymphs) submitted per geographical location were transformed to presence-only data originating 198 data points. Due to inherent limitations of modelling presence-only data, we added a similar number of data points on absence. We therefore randomly selected 200 absence and pseudo-absence points from Dataset 3 to include in Dataset 2. We recognize that other methods could be applied in the selection of the pseudo-absence points as described by [31]. However, we used the sample of absence points for Scotland that was validated by a group of entomologists and public health experts within VectorNet project [32] and offered with Dataset 3 (details below).

#### Dataset 3: “I. ricinus combined dataset”

Dataset 3 (Fig. 1c) consists of presence and absence records of *I. ricinus* (adult and nymphs) in Scotland and is part of a large dataset with *I. ricinus* records for all Europe, produced for VectorNet project [22] by a team of tick experts (a network of entomologists and public health professionals supported by the European Centre for Disease Control and Prevention, ECDC). The full methodology is described in an ECDC internal document (manuscript in preparation) [32]. In VectorNet project tick records were assembled from different sources, from public submissions (including presence-only data from Dataset 2 for Scotland) and from scientific tick surveys (including Dataset 1 for Scotland) and then validated. Due to the small amount of absence data in comparison with presence data, absence points were assigned using a mask of suitable habitats for *I. ricinus*. The habitat suitability mask was defined by the same tick experts from VectorNet project as primary, secondary and unsuitable habitat types (land classes where a species is unlikely to be found except in exceptional circumstances such as continuous and discontinuous urban fabric, industrial or commercial units, port areas, post-flooding or irrigated croplands (or aquatic) or closed to open (> 15%) broadleaved forest regularly flooded) from two land cover maps: CORINE 2006 and GLOBCOVER 2009 [33, 34] and by adding additional information about *I. ricinus* environmental limits (e.g. the fact that *I. ricinus* is only present in areas with less than 150 days of snow cover per year and where the vegetation period is greater than 145 days). Inferred absences were then extracted from unsuitable areas defined by the habitat [27, 32]. The dataset used in this study, after data management, included 1102 presence points and 1058 absence points.

For consistency between datasets, only mainland Scotland was considered. The extraction of points in mainland Scotland and the random selection of 200 absence points were conducted using ArcGIS version 10.2.2 [35].

### Georeferenced environmental data and variable selection

Ecologically relevant climatic, topographic, land cover and host-related variables for *I. ricinus* occurrence and abundance were selected. These variables were collated as GIS-based raster maps. The variables used were: (i) Moderate Resolution Imaging Spectroradiometer (MODIS) variables (monthly averages, 2001–2013): Normalized Difference Vegetation Index (NDVI, 1 km resolution), land surface temperature (LST, 1 km resolution), cumulative land surface temperature (end of May 2010–2012, MODIS derived data, 0.01 degree resolution (~1.1 km)) and length of vegetation growth period (2008–2014, 0.01 degree resolution); (ii) topographic variables: elevation above sea level (90 m resolution); (iii) long-term average climate data from UK Met Office (from 1981–2010, 5 km resolution): monthly maximum, mean and minimum temperature, number of consecutive dry days (annual average), extreme temperature range (annual average), rainfall (monthly total precipitation), days of air and ground frost (monthly average), mean relative humidity (monthly average); (iv) host-related variables: an index of presence of roe [36] and red deer [37] [both at resolution of 0.008333 degree (~ 1 km)], and red deer density for 2016 (head per square km, based on a 10 km radius smoothing of the Deer Management Unit density figures [38]); and (v) Land Cover 2006 (0.008333 degree resolution). Monthly derived variables were extracted from each month.

For data extraction compatibility and modelling purposes, all variables were converted to a standardised extent (mainland Scotland), format (tif), resolution (1 km) and projection (British National Grid). Environmental data were extracted for each of the sites of tick collection and reporting [687 sites with counts of *I. ricinus* (Dataset 1); 398 presence–absence points (Dataset 2); and 2160 presence–absence points (Dataset 3)] using the tool extract multiple values to points from ArcGIS version 10.2.2 [35].

Before model implementation, a correlation analysis and a univariate regression analysis were performed with each response variables. If a variable was strongly correlated with another variable (correlation coefficient higher than 0.6), one of them was dropped (variables with correlation coefficient between 0.5 and 0.6 were kept for analysis but under observation for possible interactions). Following with univariate analysis, biologically relevant variables with a *P*-value less than 0.10 were considered as model candidates.

In general, due to issues of autocorrelation and collinearity, satellite-derived covariates were preferred when compared with similar interpolated climatic variables [39].

### Statistical model, model validation and predictive map

Models were fitted using the Integrated Nested Laplace Approximation (INLA) R package. This Bayesian approach was selected due to its ability to account for irregular sampling intensity, spatial dependency and to quantify uncertainty in data and variables, attributing to each variable a distribution of values [40]. We recognize that other methods could be used but our objective was not to compare different modelling techniques for species distribution models, but instead compare dataset types using the same modelling technique.

The response variables were the count of *I. ricinus* ticks (nymphs and adults) per drag, site of collection and visit and *I. ricinus* (nymphs and adults) presence and absence. A model for predicting tick relative abundance was first created considering just the counts of nymphs per drag, site and visit because nymphs of *I. ricinus* pose the greatest risk of tick bites of humans [17]. However, for consistency with Datasets 2 and 3 which include higher reports of adult ticks, it was decided to model *I. ricinus* relative abundance considering the total count of adult and nymphs per drag, site and visit. This model did not differ significantly from the model using nymphs only.

The fixed effects were the previously selected set of most suitable environmental variables, including the spatial location of the data (as an interaction term between latitude and longitude). A zero-inflated Poisson distribution was chosen to model *I. ricinus* abundance (Model 1) due to a high number of survey drags with 0 counts. Presence and absence of *I. ricinus* (Model 2 and Model 3) was modelled as a binomial distribution.

The selected model for predicting *I. ricinus* relative abundance (Model 1) had two random effects: the effect of the site to capture the unstructured heterogeneity in the distribution of tick abundance among sites, and the effect of each data point (each drag) in order to account for overdispersion not captured by the zero-inflated Poisson and also to account for possible serial correlation in the data arising due to repeated sampling or drags in each site. Tick presence and absence (Model 2 and Model 3) was modelled without random effects because the inclusion of random effects did not improve model fit and predictive power.

The models were evaluated using the Deviance Information Criteria (DIC) as a measure for goodness-of-fit and a parameter from the cross-validation leave-one-out, namely the negative of the sum of the log-conditional predictive ordinance (log-CPO score) as a measure for the predictive quality of the model [41]. A backward stepwise procedure was used to select the most parsimonious model. For all three datasets, the most suitable models were selected based on the lowest values of DIC and log-CPO, amongst competing models with various covariate combinations.

The model posterior means were used to produce the predictive maps of *I. ricinus* abundance (Model 1/Dataset 1) and presence–absence (Model 2/Dataset 2 and Model 3/Dataset 3). The difference between the 97.5% and 2.5% quartiles of the predicted values were used to create uncertainty maps. The resolution of all maps was 1 km^2^ each pixel.

A matrix of boxplots, comparing the interquartile range of the models’ covariates over mainland Scotland with the interquartile range of the same covariates covered by the data points in each model, was developed.

Descriptive analyses, plots, models and maps were made using R software version 3.4.4 [42].

## Results

Figure 1 presents the spatial distribution of the three datasets of *I. ricinus* counts per drag (Dataset 1, Fig. 1a) and presence and absence (Datasets 2 and 3, respectively, Fig. 1b, c). Figure 1a shows an uneven distribution of tick collection sites over mainland Scotland, with aggregation of collection sites in the east, particularly Aberdeenshire and in opposite, lack of sampling points in the west coast. The distribution of data points in Dataset 2 (Fig. 1b) is sparse compared to Dataset 3 (Fig. 1c).

### Model 1 (tick relative abundance, using Dataset 1: quantitative survey data)

**Fig. 2:**
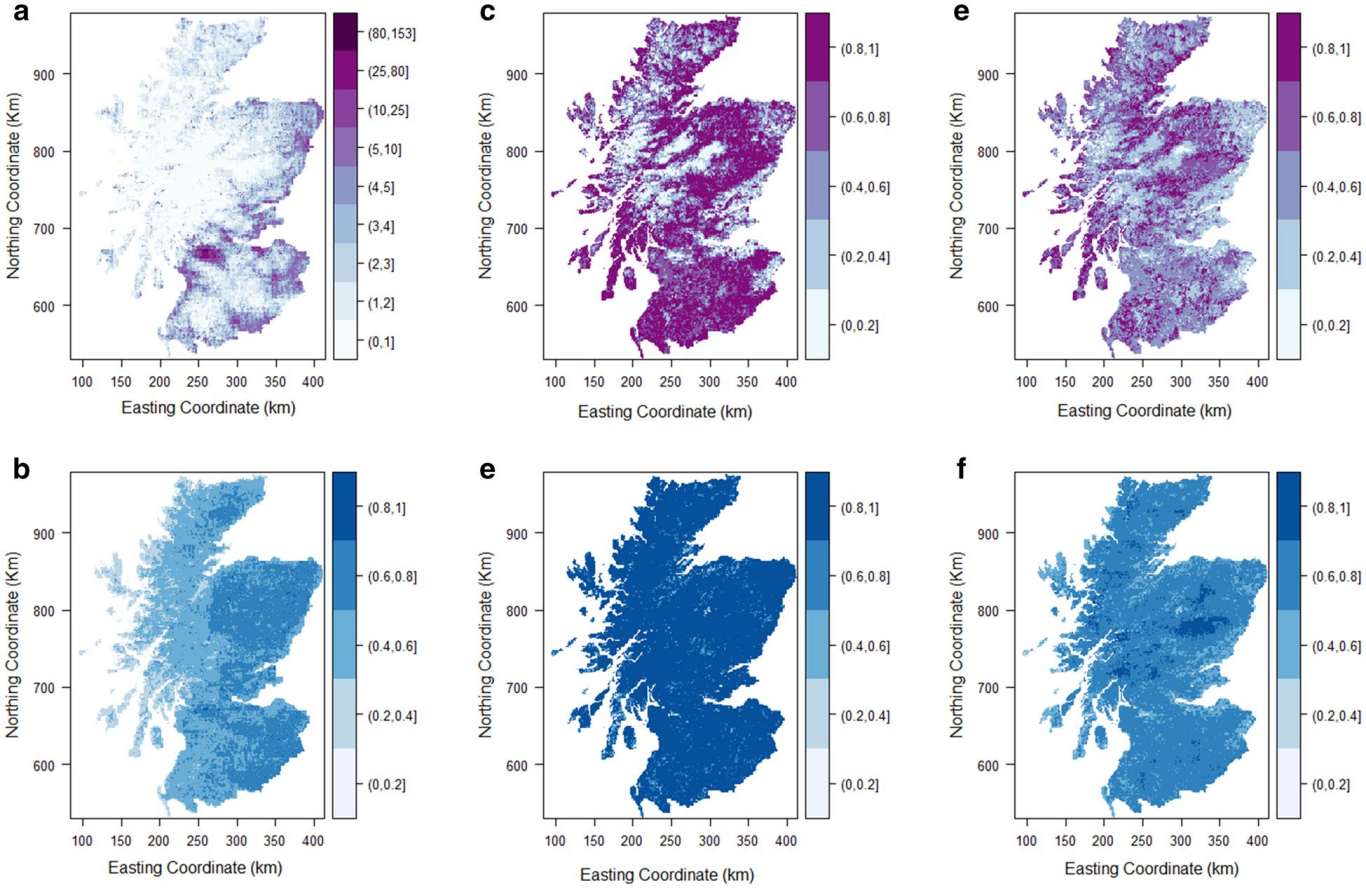
Predictive map of *I. ricinus* questing tick abundance in April in mainland Scotland (**a**) and uncertainty map (Dataset 1) (**b**); predictive map of probability of presence of *I. ricinus* using presence-only data from public submissions and absence points (**c**) and uncertainty map (Dataset 2) (**d**); predictive map of probability of presence of *I. ricinus* using the combined presence data from public submissions and tick quantitative surveys (**e**) and respective uncertainty map (Dataset 3) (**f**). The uncertainty maps were calculated from the range of 95% confidence intervals of predicted values and rescaled to a 0–1 scale. Darker areas of blue have higher uncertainty

A spatial model of the count of ticks (adult and nymphs) per drag, visit and site was run initially (DIC of the most suitable spatial model is 29786.66, log-CPO is 20427.23). Subsequently, month was added in the model as a categorical variable, improving model predictive power (DIC 29774.49; log-CPO 19686.78). The model fitted the data well (goodness-of-fit plot in Additional file 1: Figure S1). The results of Model 1 are presented in Table 1. A map for the month with highest predicted tick abundance (April) was created (Fig. 2a).

**Table 1:** Model 1: posterior mean, standard deviation, 2.5% and 97.5% quartiles and estimates of fixed and random effects for the seasonal model of tick abundance, Dataset 1

Fixed effects	Mean	SD	2.5% quartile	97.5% quartile
Intercept	− 150.7016	32.7201	− 214.9447	− 86.5187
April	2.3606	0.3390	1.7198	3.0520
May	1.9424	0.3174	1.3467	2.5944
June	1.8192	0.3178	1.2227	2.4718
July	1.2388	0.3149	0.6485	1.8863
August	1.3438	0.3151	0.7530	1.9916
September	1.4308	0.3186	0.8325	2.0850
Land surface temperature in July	0.0103	0.0022	0.0059	0.0147
No. days of frost in September	− 0.4035	0.0954	− 0.5910	− 0.2167
Roe deer	0.0096	0.0034	0.0030	0.0163
% cover of deciduous woodland	2.5341	0.7380	1.0837	3.9806
% cover of coniferous woodland	0.9053	0.2138	0.4848	1.3240
Interaction between latitude and longitude	0.0010	0.0018	− 0.0026	0.0045

*Abbreviation*: SD, standard deviation

Land surface temperature in July, presence of roe deer and deciduous and coniferous forest were associated with an increase in *I. ricinus* questing tick abundance, whilst a higher number of frost days in September lead to a decrease in tick abundance. April had the highest questing tick abundance (Table 1). The interaction term between latitude and longitude was included in the model because, although not significant, it decreased model residual variance and can help take into account spatial effects in questing tick abundance, such as spatial autocorrelation (Table 1).

The predictive map of tick abundance (Fig. 2a) shows that tick abundance increases from the north and west to the south and east of Scotland with highest predicted tick abundance in Aberdeenshire and the central belt. Areas of average to high uncertainty are present over all of the east, centre and south of Scotland whereas all the west shows a lower level of uncertainty in predicted values (Fig. 2b).

### Model 2 (tick presence–absence, using Dataset 2: tick public submissions)

Model 2 (DIC of 388.61, log-CPO of 195.81) fitted the data well (plot of model goodness-of-fit in Additional file 2: Figure S2). Presence of *I. ricinus* was correlated with an increase in NDVI and some measure of habitat composition. An increased number of days with air frost in November and increased precipitation in April were associated with tick absence. The site location of tick submission was important (Table 2). The predicted map for Model 2 (Fig. 2c) does not capture well areas of lower probability of tick presence and shows very high levels of uncertainty for most of Scotland (Fig. 2d).

**Table 2:** Posterior mean, standard deviation, 2.5% and 97.5% quartiles for the binomial models of tick presence–absence with the data from public submissions (Dataset 2) and the combined dataset (Dataset 3)

Model	Fixed effects	Mean	SD	2.5% quartile	97.5% quartile
Model 2: Presence–absence model with presence points from public submissions plus absence points	Intercept	− 6.2657	1.0232	− 8.3326	− 4.3135
	NDVI August^a^	0.1373	0.0176	0.1040	0.1732
	No. days of air frost November	− 0.1729	0.0521	0.2784	− 0.0738
	Rain April	− 0.0148	0.0053	− 0.0255	− 0.0045
	% cover of coniferous woodland	5.1989	1.2015	3.0921	7.8095
	% cover of moorland	2.2180	0.5656	1.1499	3.3725
	Interaction between latitude and longitude	0.0053	0.0036	− 0.0017	0.0123
Model 3: Presence–absence model with composite dataset	Intercept	− 3.4700	0.4771	− 4.4160	− 2.5424
	NDVI August	0.0005	0.0001	0.0004	0.0006
	Deer density	0.0336	0.0100	0.0139	0.0533
	No. days of air frost November	− 0.0527	0.0207	− 0.0936	− 0.0122
	Rain April	− 0.0123	0.0020	− 0.0163	− 0.0085
	% cover of moorland	1.3920	0.1640	1.0726	1.7161
	% cover of deciduous woodland	3.1762	0.6757	1.9203	4.5770
	% cover of coniferous woodland	2.1861	0.2128	1.7753	2.6100
	Interaction between latitude and longitude	− 0.0029	0.0013	− 0.0054	− 0.0004

^a^The posterior mean of NDVI was divided by 100

*Abbreviation*: SD, standard deviation

### Model 3 (tick presence–absence, using Dataset 3: combined dataset)

The adopted model (Model 3) gave the lowest values of DIC of 2614.61 and a log-CPO of 1307.74 (plot of model goodness-of-fit in Additional file 3: Figure S3). Model 3 presented very similar covariates as Model 2 but deciduous forest and deer density became significant predictors, likely due to the increased number of points used to model tick presence–absence (Table 2). Figure 2e shows a similar pattern of *I. ricinus* probability of presence as Fig. 2c, but the predictive map using Model 3 has more detailed definition. The uncertainty is lower for the east of Scotland and in the north and centre of the Highlands (Fig. 2f).

**Fig. 3:**
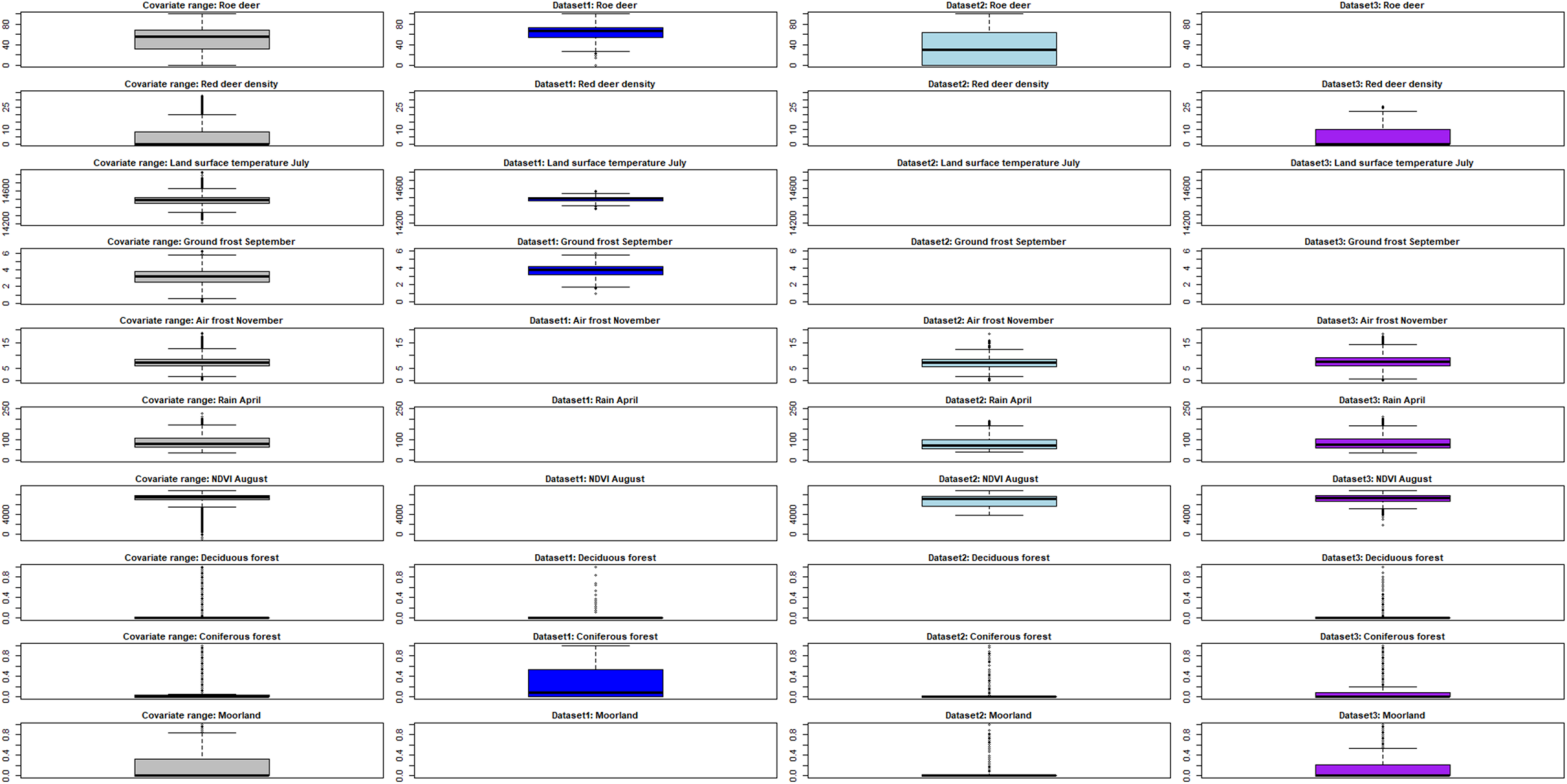
Matrix of boxplots showing the interquartile range of the covariates over mainland Scotland and compared with the range of the same covariates covered by the data points in each model

We assessed how well the three datasets cover the range of the covariates used in the models to explore the validity of the predictions. The interquartile range of each covariate in mainland Scotland was compared to the interquartile range of each covariate in the models for the data points included (Fig. 3). Although the models fitted the data well, the predictions of the three models were associated with uncertainty that was not captured in the uncertainty measures in Fig. 2, because the tick data did not cover all the range of the covariates used. Dataset 3 covered the covariate range used in the predictions better than Dataset 1 or 2. Dataset 1 was mainly collected in predominantly forest areas. The covariate index of presence of roe deer was found important for *I. ricinus* presence in Dataset 2 and it was included in the first selected Model 2 (see Additional file 4: Table S1, first model). However as can be seen in Fig. 3, the range of the covariate index of roe deer presence is not well covered by Dataset 2, contributing to higher uncertainty in the predictions (see Additional file 5: Figure S4a, b). The covariate roe deer was therefore removed from the final model (Table 2). Using all the covariates of Model 3 for fitting a model with Dataset 2 helped to corroborate how the covariates (type and range) are important in predictive mapping and can be a source of error for model predictions (also shown in Additional file 4: Table S1 and Additional file 5: Figure S4c, d).

## Discussion

Predictive maps of tick distribution are essential for understanding human disease risk and allocating resources for prevention and control. However, they require extensive data on tick distribution, and robust long-term and geographically extensive datasets are often difficult to obtain. Therefore datasets are often used that were not collected for this purpose, or do not cover the entire extent of the required predicted distribution. The main aim of this study was to compare the performance of three different types of data to predict *I. ricinus* distribution in Scotland. We critically assessed modelled distributions to conclude how useful each dataset may be to inform policy, future data collection and risk mapping, both for Scotland and more widely.

### Predictors for *I. ricinus* relative abundance and presence–absence

The predictors identified in the models are consistent with the current knowledge on *I. ricinus* ecology. *Ixodes ricinus* activity is seasonal, and in Scotland peaks in April and then generally declines over the season as ticks die or find a host (Model 1). It is not surprising that roe deer presence and red deer density are correlated with both tick abundance and presence as deer are important hosts maintaining *I. ricinus* populations [11]. Many other studies have found deer abundance as a predictor of tick presence and/or abundance [11, 29, 43]. Temperature affects tick behaviour, interstadial development rate, fertility, survival and the proportion of active ticks [12–14]. Warmer climates are frequently associated with tick presence and abundance (e.g. [28]). Land surface temperature was also used in other studies to estimate *I. ricinus* presence–absence and abundance [39, 44]. In accordance, in this study we found that areas with warmer climates and lower number of frost days (minimum temperature below 0 °C) were associated with higher tick abundance, consistent with previous studies [25, 45].

Forested areas, particularly mixed and deciduous forest, as well as other habitats that provide a sheltered canopy over the ground, such as bracken and deep heather in moorland that are characterized by thick ground vegetation or shrub and deep leaf litter layers, provide moist and mild microclimates which aid tick survival and activity. Forests are also a source of food and shelter for many species of tick hosts [46]. NDVI, which quantifies the level of photosynthetic activity of the vegetation, has been previously identified as being an important physical parameter that correlates with *I. ricinus* presence and abundance [39]. More questing *I. ricinus* are predicted in areas with warmer climate and higher rainfall or higher humidity [47]. However, the negative effect of annual precipitation that we found in this study, was also found by James et al. [29] in Scotland and Schulz et al. [48] in Germany. These findings suggest that the wettest conditions in Scotland are probably too wet for *I. ricinus* to quest, while drier areas of Scotland are still wet enough for good survival rates.

For all three datasets plausible predictors were identified and predictive maps were created. Although the models fitted the data well, the three predictive maps do not present a consistent pattern of *I. ricinus* distribution and the predictions are associated with a large amount of uncertainty, particularly for Model 2 and to a lesser degree for Model 1. The uncertainty presented in Fig. 2 (b, d, f; difference between 2.5% and 97.5% values), provides a measure of the predicted uncertainty at each pixel. However, the predicted uncertainty is correlated with the model posterior mean for each covariate. In addition, this measure does not include uncertainty associated with extrapolation outside the covariate range. We therefore explored factors that could influence the validity of the predictions for each dataset, and identified when each dataset might be most appropriately used.

### Use of quantitative *I. ricinus* survey data (Dataset 1)

The relative abundance of questing *I. ricinus* ticks is generally measured by dragging a blanket over the vegetation. This technique does not measure the absolute density of the whole tick population in an area because it does not count ticks that are moulting, resting, feeding or in diapause [44]. It is also worth noting that the blanket drag method’s efficiency is affected by ground vegetation height and density [43]. Ground vegetation height and density should be included in statistical models and repeated sampling of a site is recommended due to the impact of weather conditions on tick activity on the day of sampling. Another limitation from this technique is that tick absence data cannot be considered completely free of error because some of the zeros could in reality indicate very low tick densities rather than true absolute absence, due to the finite number of blanket drag transects per site and conditions on the day of surveying [44]. However, this is a standard scientific technique which provides a useful comparable index of abundance of questing nymphs between sites [22].

Conducting blanket drags is time-consuming which makes it resource-intensive to perform large-scale, long-term field studies using this technique. However, estimating questing *I. ricinus* relative abundance gives more information about this species distribution when compared with presence-only and presence–absence data. Abundance data are necessary to calculate the density of infected ticks, which is important in estimating disease risk [44], as well as providing more information on *I. ricinus* dynamics. Abundance data also improve model accuracy, predictive performance and ability to discriminate trends at finer scales, compared to presence–absence data. This improvement is particularly important for species of high abundance compared to “rare” species [49]. When the objective is to create a predictive map for a country such as Scotland where *I. ricinus* is endemic, abundance models will provide more meaningful distribution maps.

The predictive map of questing *I. ricinus* relative abundance enables us to clearly identify areas with high and low tick abundance, and shows an increasing trend of tick abundance from the west to the east coast of Scotland. However, although the predictions of relative abundance of questing ticks had lower uncertainty compared to both of our predictive maps of *I. ricinus* probability of presence, it is clear that the sample sites are clustered and do not cover all of mainland Scotland, and that the covariate range covered by mainland Scotland is not fully represented in the data. Since there is an ecological gradient from the west (higher temperatures, higher rainfall) to the east, it is concerning that there may be insufficient data for accurate predictions in the west. Dataset 1 was collected mainly (although not exclusively) in forest areas, reflecting the data collection, which was aimed at specific ecological studies [29, 30]. Hence the dataset does not have good coverage for some areas of mainland Scotland and therefore the reliability of the predictions is like to be lower outside the core survey areas. These issues of low coverage are common to these type of data, due to the resources needed to collect quantitative survey data over a wide scale. Quantitative survey data are often considered the “gold standard”, but this is only true for models that make predictions in the same geographical area and covariate range from which the surveys were conducted.

### Use of *I. ricinus* presence data obtained through public submissions plus absence points (Dataset 2)

Presence-only data, generated from submissions by the public and often obtained from citizen science studies, are frequently used to map species distribution. These data usually require fewer resources to collect than scientific surveys [50] but include random error associated with uncertainty in the location of ticks were collected, variability in sampling (e.g. variability between different people reporting) and in effort (e.g. some people contribute more data than others and effort can also change over time) [50]. Bias is also associated with the fact that people report from places that are visited frequently or are more accessible [51]. This type of data lacks information on where the species is absent, which limits the predictive power of the inference and also restricts the type of questions that can be asked [52]. In this study, information of where the tick was absent (true absences from the scientific surveys from Dataset 1 and pseudo-absence points from habitat unsuitability mask from Dataset 3) was added to the presence-only records from submissions to improve the predictive power of the model. This process is not free of error since *I. ricinus* is not confirmed to be absent at all the points used as absence [52]. A general disadvantage of this type of distribution data is that all presences are treated as equal, regardless of the abundance of *I. ricinus* ticks that the habitat supports, which may not provide enough information to enable the model to differentiate a scarce habitat classified as having the species present from a habitat where the species is in fact established [49].

The predictive map resulting from Model 2 presents lower spatial detail compared to the other two maps, indicates high probability of *I. ricinus* presence over much of mainland Scotland and does not reflect the known vector habitat preferences, as presence is predicted in some unsuitable areas. This does not provide particularly useful information for targeting public health interventions and illustrates the challenges of using sparse presence–absence data in areas where ticks are endemic. The predictive map presents high uncertainty demonstrating low confidence in the predictions, likely due to the small sample size. In addition, there is uncertainty relating to the predictions as Dataset 2 does not cover all the covariate range (Fig. 3).

Although the potential biases of submission data are common to similar studies, they can often be minimised if sufficient sample sizes are obtained. The dataset used in this survey was not collected for the purpose of mapping tick distribution, so the sample size was low (~ 200). In England and Wales where the submission scheme has been promoted, over 4000 data points were collected for the same period, giving more capacity for predictive mapping.

These results should not rule out the use of data from public submissions that can be used to infer range limits of *I. ricinus* after careful analysis to account for adventitious ticks dispersed by hosts [32].

### Use of *I. ricinus* combined datasets (Dataset 3)

The predictive model based on a dataset that combined data from scientific studies, public submissions data and absences of *I. ricinus* increased the spatial coverage of the data in mainland Scotland (Fig. 1c) and produced a more detailed predictive map. In addition this dataset had the best coverage for the covariate range used in predictions (Fig. 3). It is not surprising that the spatial trend of the predictions from both presence–absence models were comparable. However, the model developed using the combined data (Model 3) provides a better description of the presence and absence of *I. ricinus* not only because of the higher number of points but also because it includes presence and absence data from quantitative tick surveys (Dataset 1). This method of adding information from different datasets can be more easily applied at country and continental levels to obtain distribution maps. However, because composite datasets combine different types of data, it is more challenging to understand how the different errors, bias and limitations of each dataset might affect the model outputs and the predicted uncertainty.

### Predictive *I. ricinus* maps for Scotland

The three datasets used in this study are the only *I. ricinus* datasets that are available at a national scale in Scotland (as far as the authors are aware). As discussed above, although quantitative survey data are usually regarded as a gold standard, the data used in this study did not have good coverage, both geographically and over the covariate range, for the whole of Scotland. This dataset is appropriate for making decisions that require detailed distribution data only in areas where the coverage is good. Outputs from Dataset 2, comprising public submission data, were limited by the small sample sizes in this dataset, which gave high model uncertainty. Therefore, Dataset 3, which uses data from multiple sources, provides the most convincing predictive map and is recommended for decision-making at national scale.

It is conceivable that any of these maps could be used alone for decision-making, without further consideration of the limitations of the data inputs. The differences between the three maps highlight the importance of exploring sources of uncertainty in models and in predictions and presenting this alongside predictive maps. Although there are a high number of published papers on *I. ricinus* predicted distribution, uncertainty is rarely presented (a rare example is [18]). For other vectors, when uncertainty is considered, the uncertainty metric used in this study is commonly reported (such as [53]), but its value is limited because the uncertainty values correlate with the posterior mean. In addition, this measure does not include uncertainty associated with extrapolation outside the covariate range. Further development of methodological approaches to quantify this uncertainty, such as statistical tools for the diagnosis of model prediction reliability or to limit predictions to the range and covariates encountered during surveys would be beneficial [54].

Although we conducted this exercise with the aim of improving tick distribution and Lyme borreliosis management, the findings are relevant to other vector-borne disease systems for animal and human health.

## Conclusions

The choice of the most suitable model and map of *I. ricinus* distribution in Scotland depends on the objective. For local-level decision-making, Model 1 and map 1 (using quantitative *I. ricinus* survey data, Dataset 1) are more appropriate, with a good coverage for the east coast of Scotland. For decision-making at national level, Model 3 and map 3 (using combined Dataset 3) provide a better coverage of the country and the range of the covariates. Although tick surveys provide detailed data on questing tick relative abundance, the resources required often limit the number of areas that can be sampled, which makes it challenging to make predictions for extensive areas. If available at larger spatial and temporal resolution, relative abundance data will result in finer scale maps that are more effective for risk management and communication at national and regional levels. The analysis in this study highlights the need for additional surveying in areas with poor previous coverage. Future maps of *I. ricinus* abundance could be improved by adding *a priori* information of habitat preferences into the model structure [54]. For large-scale mapping at lower resolution, or if there are few tick data from quantitative surveys, data on *I. ricinus* presence-only should be combined with data from field surveys and absence data for modelling presence–absence. To overcome the problems inherent in the use of presence-only data from public submissions, it is necessary to decrease associated errors and bias by accounting for observer effort and expertise [55] or to find approaches by which absence data are also reported [44]. When predictive maps are needed for public health decision making, such as allocation of resources for awareness campaigns, information on uncertainty should be included with vector distribution maps. However, because map uncertainty reflects a single source of uncertainty (the spatial model), improved statistical techniques are required to quantify uncertainties relating to predictions.

## Supplementary information

**Additional file 1: Figure S1.** Plot of fitted (blue line) versus observed values (dots) for the seasonal model of nymph and adult abundance, Dataset 1. The observed number of nymphs plus adults per drag has a minimum of 0, a mean of 2.34 and a maximum of 109. The predicted number of nymphs plus adults per drag has a minimum of 0, a mean of 2.5 and a maximum of 106.

**Additional file 2: Figure S2.** Goodness of model fit, model 2, Dataset 2. The plot is presented as a histogram and curve for binomial regression.

**Additional file 3: Figure S3.** Goodness of model fit, model 3, Dataset 3. The plot is presented as a histogram and curve for binomial regression.

**Additional file 4: Table S1.** Posterior mean, standard deviation, 2.5% and 97.5% quartiles for the binomial models of tick presence–absence with the data from public submissions (Dataset 2), first model selected based on the Bayesian criteria; secondly using covariates from model 3 to predicted Dataset 2 distribution. None of these two models were selected for the predictions of Dataset 2.

**Additional file 5: Figure S4.** Predictive maps of binomial models of tick presence–absence with the data from public submissions (Dataset 2): predictive map from first model selected based on the Bayesian criteria (a) and uncertainty map (b); predictive map using covariates from Model 3 to predicted Dataset 2 distribution (c) and respective uncertainty map (d).

## Data Availability

Data supporting the conclusions of this article are included within the article and its additional files. The code is available *via* Zenodo: 10.5281/zenodo.3476288. Dataset 1 is available on request; please contact Professor Lucy Gilbert (Lucy.Gilbert@glasgow.ac.uk). Dataset 2 is freely available. Data from Public Health England can be download at https://nbnatlas.org/. VectorNET data (Dataset 3) is available on request from ECDC. The request needs to include the purpose of the data request. More information available at https://ecdc.europa.eu/en/publications-data/european-surveillance-system-tessy. Any question, please contact: data.access@ecdc.europa.eu.

## Abbreviations

PHE: Public Heath England
ECDC: European Centre for Disease Control and Prevention
GIS: geographical information system
MODIS: moderate resolution imaging spectroradiometer
NDVI: normalized difference vegetation index
LST: land surface temperature
INLA: integrated nested Laplace approximation
DIC: deviance information criteria
CPO: conditional predictive ordinance
