# Supplementary figures and images for: Using imperfect data in predictive mapping of vectors: a regional example of Ixodes ricinus distribution
Source: Parasit Vectors. 2019 Nov 14;12:536. doi: 10.1186/s13071-019-3784-1 (PMC6857280; doi:10.1186/s13071-019-3784-1)

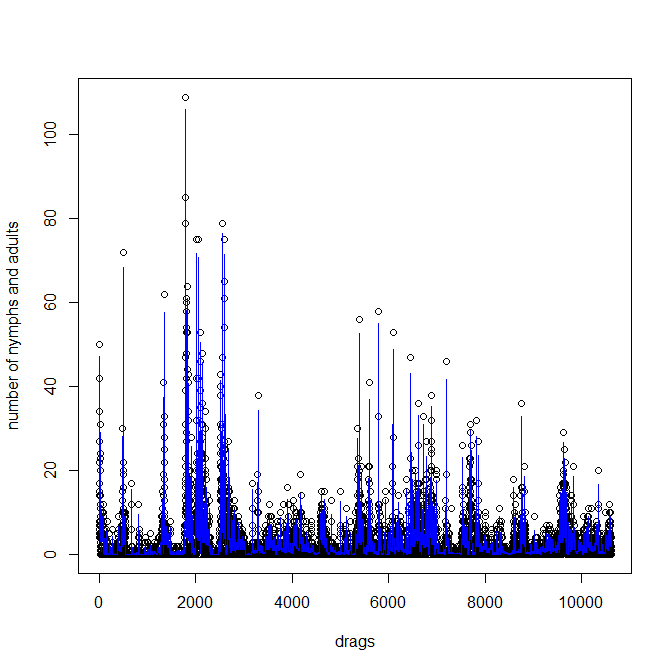

Supplement: Supplementary file 1 — Additional file 1: Figure S1. Plot of fitted (blue line) versus observed values (dots) for the seasonal model of nymph and adult abundance, Dataset 1. The observed number of nymphs plus adults per drag has a minimum of 0, a mean of 2.34 and a maximum of 109. The predicted number of nymphs plus adults per drag has a minimum of 0, a mean of 2.5 and a maximum of 106. [file 13071_2019_3784_MOESM1_ESM.tif]

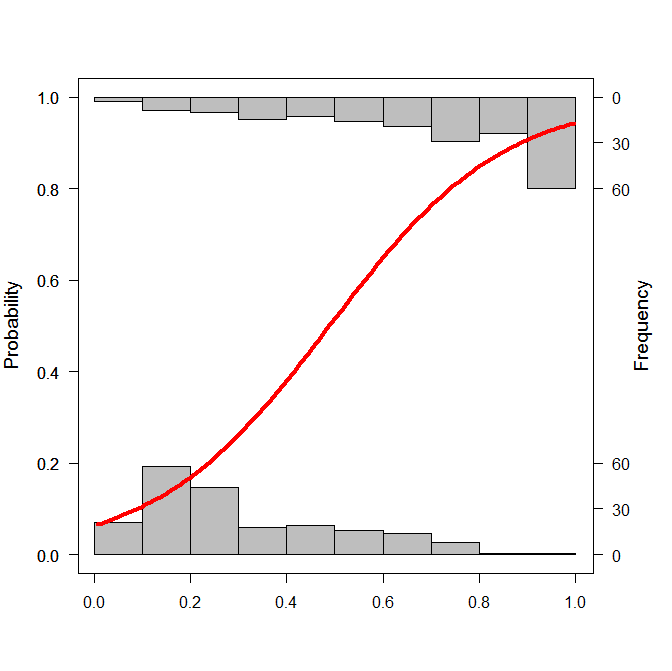

Supplement: Supplementary file 2 — Additional file 2: Figure S2. Goodness of model fit, model 2, Dataset 2. The plot is presented as a histogram and curve for binomial regression. [file 13071_2019_3784_MOESM2_ESM.tif]

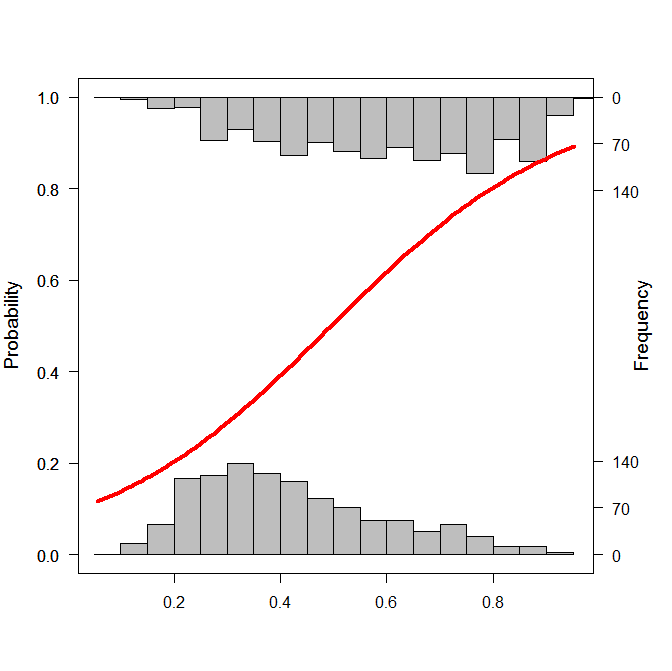

Supplement: Supplementary file 3 — Additional file 3: Figure S3. Goodness of model fit, model 3, Dataset 3. The plot is presented as a histogram and curve for binomial regression. [file 13071_2019_3784_MOESM3_ESM.tif]

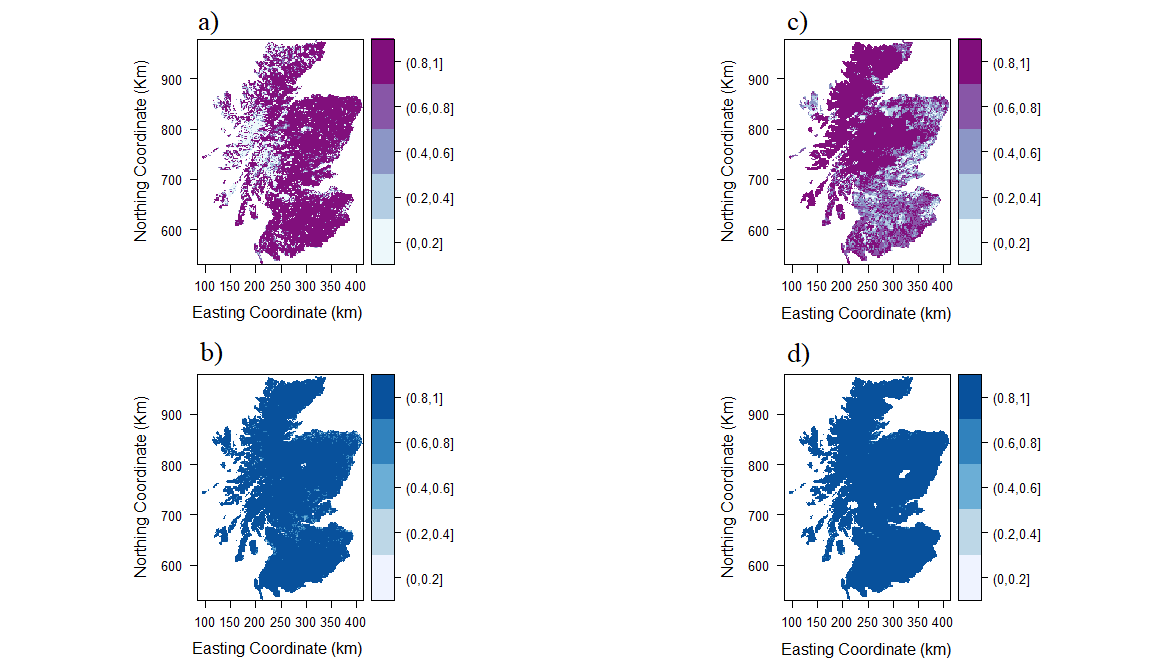

Supplement: Supplementary file 5 — Additional file 5: Figure S4. Predictive maps of binomial models of tick presence–absence with the data from public submissions (Dataset 2): predictive map from first model selected based on the Bayesian criteria (a) and uncertainty map (b); predictive map using covariates from Model 3 to predicted Dataset 2 distribution (c) and respective uncertainty map (d). [file 13071_2019_3784_MOESM5_ESM.tif]
