# Supplementary material for: Using imperfect data in predictive mapping of vectors: a regional example of Ixodes ricinus distribution
Source: Parasit Vectors. 2019 Nov 14;12:536. doi: 10.1186/s13071-019-3784-1 (PMC6857280; doi:10.1186/s13071-019-3784-1)
Supplement: Supplementary file 4 — Additional file 4: Table S1. Posterior mean, standard deviation, 2.5% and 97.5% quartiles for the binomial models of tick presence–absence with the data from public submissions (Dataset 2), first model selected based on the Bayesian criteria; secondly using covariates from model 3 to predicted Dataset 2 distribution. None of these two models were selected for the predictions of Dataset 2. [file 13071_2019_3784_MOESM4_ESM.docx]

**Additional file 4: Table S1.** Posterior mean, standard deviation, 2.5% and 97.5% quartiles for the binomial models of tick presence-absence with the data from public submissions (dataset 2). The first model was selected based on the Bayesian criteria; the second model was fitted using the significant covariates of model 3. None of these two models were selected for the predictions of dataset 2.

| **Model** | **Fixed effects** | **mean** | **standard deviation** | **2.5% quartile** | **97.5% quartile** |
| --- | --- | --- | --- | --- | --- |
| Model 2: Presence-absence model with presence points from public submissions plus absence points. This model was selected based on DIC and CPO criteria. | Intercept | -4.9576 | 1.8414 | -8.6884 | -1.4563 |
|  | NDVI August^a^ | 0.1504 | 0.0296 | 0.0956 | 0.2120 |
|  | Roe deer | 0.1234 | 0.0128 | 0.1001 | 0.1506 |
|  | No. days of air frost November | -0.4190 | 0.1195 | -0.6631 | -0.1939 |
|  | Rain April | -0.0280 | 0.0089 | -0.0462 | -0.0111 |
|  | % cover of moorland | 2.9543 | 1.0542 | 0.9449 | 5.0844 |
|  | Interaction between latitude and longitude | 0.0166 | 0.0065 | 0.0042 | 0.0297 |
| Model 2: Presence-absence model with presence points from public submissions plus absence points. This model was fitted with the significant covariates used in model 3 (combined dataset). | Intercept | -3.8516 | 1.1225 | -6.1032 | -1.6939 |
|  | NDVI August | 0.0012 | 0.0002 | 0.0008 | 0.0016 |
|  | Deer density | 8.3610 | 11.4365 | -8.1275 | 35.2416 |
|  | No. days of air frost November | -0.2962 | 0.0749 | -0.4506 | -0.1563 |
|  | Rain April | -0.0199 | 0.0074 | -0.0350 | -0.0057 |
|  | % cover of moorland | 0.4616 | 0.8325 | -1.2237 | 2.0459 |
|  | % cover of deciduous woodland | 26.5532 | 15.9831 | 0.7997 | 62.9048 |
|  | % cover of coniferous woodland | 5.5018 | 1.2241 | 3.3527 | 8.1592 |
|  | Interaction between latitude and longitude | 0.0076 | 0.0041 | -0.0003 | 0.0157 |

^a^In this model the posterior mean of NDVI was divided by 100.
